# Communities as cliques

**DOI:** 10.1038/srep35648

**Published:** 2016-10-19

**Authors:** Yael Fried, David A. Kessler, Nadav M. Shnerb

**Affiliations:** 1 Department of Physics, Bar-Ilan University, Ramat-Gan IL52900, Israel

## Abstract

High-diversity species assemblages are very common in nature, and yet the factors allowing for the maintenance of biodiversity remain obscure. The competitive exclusion principle and May’s complexity-diversity puzzle both suggest that a community can support only a small number of species, turning the spotlight on the dynamics of local patches or islands, where stable and uninvadable (SU) subsets of species play a crucial role. Here we map the question of the number of different possible SUs a community can support to the geometric problem of finding maximal cliques of the corresponding graph. This enables us to solve for the number of SUs as a function of the species richness in the regional pool, *N*, showing that the growth of this number is subexponential in *N*, contrary to long-standing wisdom. To understand the dynamics under noise we examine the relaxation time to an SU. Symmetric systems relax rapidly, whereas in asymmetric systems the relaxation time grows much faster with *N*, suggesting an excitable dynamics under noise.

Competition is ubiquitous in nature. Almost any aspect of living systems, from the molecular level to ecological scales, involves the competition of different species for a finite set of resources. As different populations grow, resource levels decline, putting stress on other individuals and leading to extinction of some forms of life and to saturation of others, generating the observed patterns of life on all timescales. Accordingly, the interplay between competitive exclusion^1^,^2^, ecosystem’s complexity^3^,^4^ and the biodiversity plays a central role in the theory of community dynamics.

Still, many fundamental aspects of the theory of competition and its applicability to empirically observed patterns are far from being understood. The competitive exclusion principle^5^,^6^ predicts that the maximum number of species allowed in a local community is smaller or equal to the number of limiting resources, in apparent contrast with the dozens and hundreds of species of freshwater plankton^1^,^2^, trees in tropical forests^7^ and coral reef^8^. May’s complexity-diversity analysis^3^,^4^ presents another level of difficulty; it states that, even when the number of resources is large enough, a substantial niche-overlap between species makes the chance of stable coexistence exponentially small in *N*, the species richness of the community. These long standing puzzles have received a lot of attention over the last decades, with many mechanisms suggested to circumvent the mathematical constraints and many works that have tried to provide empirical support to these theories^9^. Nevertheless, there seems to be no general, well established and confirmed theory that explains the persistence of high-diversity assemblages.

While some communities that support biodiversity may be considered as well-mixed, generically these communities have a spatial structure. The dynamics takes place in local habitat patches, connected to each other by migration. Different realizations of this scenario, ranging from the McArthur-Wilson mainland-island model (a single and relatively small patch is coupled to a well-mixed large system)^10^,^11^ to the conceptual framework of metapopulations and metacommunities (a system of many, diffusively coupled, local patches)^12^,^13^ have been considered in the literature.

Whichever version of these spatially structured dynamics one adopts, one immediately encounters a fundamental problem: to identify the assembly rules of local communities^14^,^15^ and the factors that govern their stability^16^,^17^,^18^. In the presence of strong environmental filtering one expects a one-to-one match between environment and community, rendering the effect of interspecific competition insignificant. Here we focus on the opposite scenario, where species composition is determined predominately by competition, hence the system may support multiple steady states, and different historical sequences of species entering the local community may lead to different long-term compositions. Theory, experiments and field studies focusing on the possibility of alternative steady states (and the theory of catastrophic shifts associated with this scenario) play a central role in contemporary community dynamics literature^19^,^20^.

To endure over intermediate/long timescales, a subset of size *S* < *N* species should be intrinsically stable and uninvadable. If this subset is by itself unstable the local dynamics will drive some of the *S* species to extinction. Even an intrinsically stable subset may still be invaded by one of the *N* − *S* species from the regional pool, rendering it (for reasonable values of invasion rate) a short-lived transient. A stable and uninvadable (SU) local community, on the other hand, will persist until one species goes extinct due to demographic noise or environmental variations. One then expects that the dynamics of a spatial system is dominated, on intermediate/long timescales, by SU configurations.

This insight points to a crucial question: how many SU configurations are possible, and in particular, how does this number scale with *N*? This problem has a long history. Gilpin and Case^21^ analyzed it numerically using a simple and generic description of such a community, the generalized Lotka-Volterra equations:


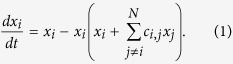


here *x*_*i*_ is the local density of the *i*-th species population and *c*_*i*,*j*_ is a zero-diagonal matrix of positive numbers, indicating the level of competition between the *i*-th and *j*-th species. The larger *c*_*i*,*j*_ is, the stronger is the stress that individuals of species *j* put on individuals of species *i*. The SU problem, formulated for this model, is then how many size *S*-subsets of the *N* species satisfy the following two conditions:

1. Stability and feasibility: Eq. 1, when limited to a size *S* subset, 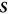, yields a time independent solution for which 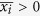 for all of the species in 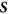, where 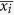 is the equilibrium density of the *i*-th species in the subcommunity.
2. Uninvadability: Eq. 1, when applied to all absent *N* − *S* species and linearized around the fixed point 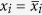 for 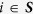 and *x*_*i*_ = 0 for 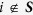, yields negative growth rates 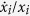 for all 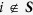.

Based on their (quite limited, by today’s standards) numerical simulations, Gilpin and Case concluded that the number of SU states grows exponentially with the richness of the regional community *N*.

Fisher and Mehta^22^, in a new work, touched on the same problem from another perspective, the niche-neutral debate and the intermediate models suggested to bridge between these opposing approaches^23^,^24^. They have heuristically mapped a variant of the competing species model to a known physical model for glassy behavior, the random energy model^25^. Armed with this mapping, Fisher and Mehta interpreted the freezing transition, associated with the temperature below which a liquid shows glassy features, as the transition between niche-like and neutral-like ecological dynamics. The weak noise regime of the Lotka-Volterra dynamics was argued to correspond to the glassy phase, where local energy minima (in physical glasses) or SU states (in ecosystems) govern the dynamics, and the system spends most of its time close to one of these attractive states until it is kicked, stochastically, to another domain of attraction. Under strong noise, on the other hand, the SU/local minima structure would be washed away by the noise, rendering a neutral-like behavior.

## The geometry of Competition

Both the Gilpin-Case and Fisher-Mehta works faced a major technical obstacle: given the interaction matrix *c*_*i*,*j*_, it is quite difficult to find all its SU states. To do that for a system with *N* species, one should scan through all the 2^*N*^ possible configurations, checking for stability/feasability and uninvadablity for each of these combinations. Therefore, Gilpin and Case considered only limited regional pools with *N* ≤ 14, while Fisher and Mehta extracted analytic results only for their simplified, presence-absence model, that was mapped to the random energy model using strong (and not necessarily realistic) assumptions regarding the connection between invasion rates and the strength of stochasticity. To proceed, we suggest a geometric reduction of the problem.

We consider first the symmetric version of the model. In this version, studied by both^21^,^22^ of the above, *c*_*i*,*j*_ = *c*_*j*,*i*_. This symmetric scenario provides more transparent results, and we will use this case to clarify the general method.

As explained in Refs 22 and 26, the interaction matrix *c*_*i*,*j*_ may be characterized by two parameters: the average value of an element 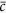 (overline denotes an average over all the *N*(*N* − 1) nondiagonal entries), reflecting the average pressure one species extracts on the other, and the variance, 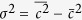.

The main insight in this work is the realization that the problem of finding the SUs simplifies tremendously in the extreme case where the competition matrix elements are either zero or very large. In a symmetric model this condition implies that every pair of species, *i* and *j*, are either

1. **non-interfering**, meaning that *i* and *j* do not compete at all and so are mutually invasible, like the different species in the McArthur-Wilson model^10^,^11^.
2. **mutually exclusive**, i.e., they cannot live together in the same local community, and none of them can invade a subcommunity that includes its opponent^14^.

For a non-interfering pair we assume *c*_*i*,*j*_ = *c*_*j*,*i*_ = 0. For a mutually exclusive pair we take *c*_*i*,*j*_ = *c*_*j*,*i*_ to be a sufficiently large number. At this point we cannot specify this large number so we use the notation *c*_*i*,*j*_ = *c*_*j*,*i*_ = ∞, meaning that the interaction is strong enough to make these species mutually exclusive. Below we will provide a lower limit for the interaction strength.

**Figure 1:**
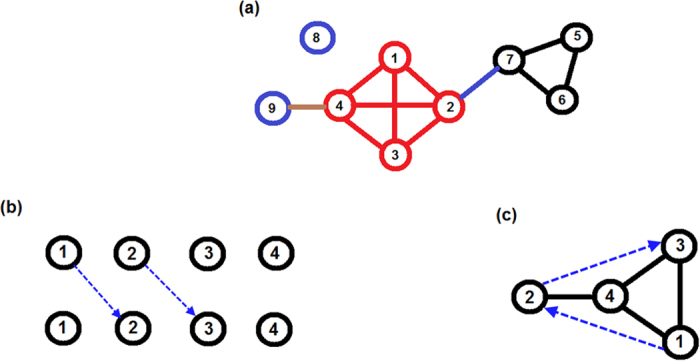
The geometric interpretation of competition networks. In (**a**) an example of a network for the symmetric model is presented. Every pair of non-interacting species is connected by a full line; for example, species 1, 2, 3 and 4 are all non-interacting, meaning that *c*_1,2_ = *c*_1,3_ = *c*_1,4_ = *c*_2,4_ = 0. A clique, like {1, 2} or {5, 6, 7}, is a noninteracting subset of the species. A clique is stable only if another species cannot invade it, so {1, 2} is unstable, since it may be invaded by 3 and 4. The stable and uninvadable subsets are the maximal cliques {1, 2, 3, 4}, {5, 6, 7}, {4, 9}, {2, 7} and {8}. (**b**) Provides an example of an asymmetric system, where a dashed line represent dominance relationships. Here species 1 dominates 2 (*c*_1,2_ = 0, *c*_2,1_ = ∞) and species 2 dominates 3. In (**c**) we present this system as a network, where full lines indicate, as before, no interaction and dashed lines with arrows indicate dominance, the arrow pointing towards the inferior species. Although {1, 3, 4} and {2, 4} are both maximal cliques, only {1, 3, 4} is SU (2 cannot invade since 1 dominates it) while {2, 4} is invadable by 1.

In Fig. 1 we show how to represent such a scenario by a network. First, every species is represented by a vertex, and the *i* and *j* vertices are linked (adjacent) iff the corresponding species are non-interfering. Accordingly, the *i, j* element of the adjacency matrix of this undirected graph is unity if *c*_*i*,*j*_ = 0 and zero if *c*_*i*,*j*_ = ∞.

Given this representation, the one to one correspondence between SU subcommunities and *maximal cliques* of the corresponding graph is easily recognized. A clique is a collection of vertices that are all connected, every one to each of the others, here representing a group of species that can all coexist. To be uninvadable this clique should be maximal, i.e., a clique that cannot be extended by including any other adjacent vertex. Although the problem of finding all maximal cliques of a graph is known to be NP-complete^27^, in practice there are efficient algorithms^28^ that allow one to find cliques quite easily for *N* up to 500, way above the numbers that have been previously considered in the SU context.

More importantly, this simple geometric interpretation of the problem allows us to also obtain analytic results. The expected number of maximal cliques with exactly *S* species, *SU*(*N, S*), may be determined by multiplying the number of subsets of size *S* (binomial factor) by the chance that a single, randomly chosen subset is indeed a maximal clique. For random symmetric interactions the adjacency graph is an Erdös-Renyi network of size *N*, and the result is given by^29^,


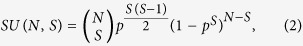


where *p* is the probability that two randomly chosen vertices are connected. In section I of the Supplementary Information (SI) we show how to find an asymptotic expression for this sum using Laplace’s method,


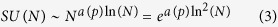


where *a*(*p*) = 1/[2 ln(1/*p*)].

**Figure 2:**
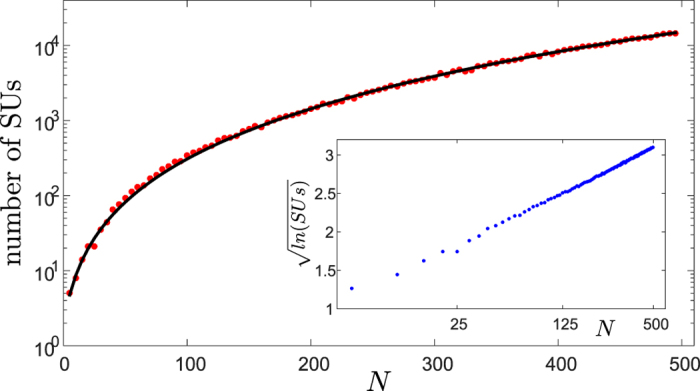
Number of maximal cliques as a function of *N* for the symmetric zero-infinity model, plotted on semilog-y scale. Results were obtained from a symmetric model with *p* = 0.1, *N* running from 5 to 500 in intervals of 5. Points correspond to the number of maximal cliques in a single realization, full line is *N*^0.221 log(*N*)^. In the inset we plot 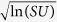 on semilog-x scale, emphasizing that this is a straight line, in agreement with Eq. 3.

Figure 2 shows the increase of *SU* with *N*, with perfect agreement with Eq. (3). This by itself is a counter-example to the central conclusion of Ref. 21 and to the main outcome of Ref. 22: the number of SU states does *not* increase exponentially with *N*. Therefore, the analogy suggested^22^ between the random energy model, and the problem at hand turns out to be problematic. Even if there is a direct thermodynamical analog to the community dynamics problem, where SUs are mapped to local minima and the effect of noise is equivalent to temperature, one would have to be a variant of the random energy model with *N*^*aln*(*N*)^ number of states, as opposed to 2^*N*^ in its standard spin version. In such a case the temperature of the glass transition (as before, this is analogous to the level of noise below which the system is niche-dominated, and above which the dynamics appears to be neutral) diverges like *N*/ln^2^(*N*) at large *N*, meaning that there is no true neutral phase, and the system is niche dominated even if the noise is relatively large (see discussion section).

Now let us consider the more general case, where the interaction matrix has no symmetry properties but the *c*_*i*,*j*_s are still either infinite or zero. Any pair of species may be in one out of three relationships: mutually exclusive (*c*_*i*,*j*_ = *c*_*j*,*i*_ = ∞), non-interfering (*c*_*i*,*j*_ = *c*_*j*,*i*_ = 0) or dominance (*c*_*i*,*j*_ = ∞, *c*_*j*,*i*_ = 0, meaning that *j* is superior to *i*). Figure 1b provides a demonstration of these possibilities.

To interpret this scenario geometrically, one needs two types of links. First, every two non-interfering nodes are connected with undirected links (full lines in Fig. 1c), as in the symmetric case. Second, any pair of nodes that admit dominance relationships are connected by a different type of directed link (a dashed line, with an arrow pointing towards the inferior species, in 1c). A stable subcommunity in this case is a clique of non-interacting species as before, but the condition for uninvadablity is different. It is not enough for a clique to be maximal; to be an SU it should fulfill another requirement, namely, that every node not in the clique should be dominated by at least one species in the clique. The expected number of such cliques is then,


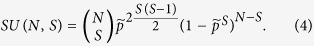


**Figure 3:**
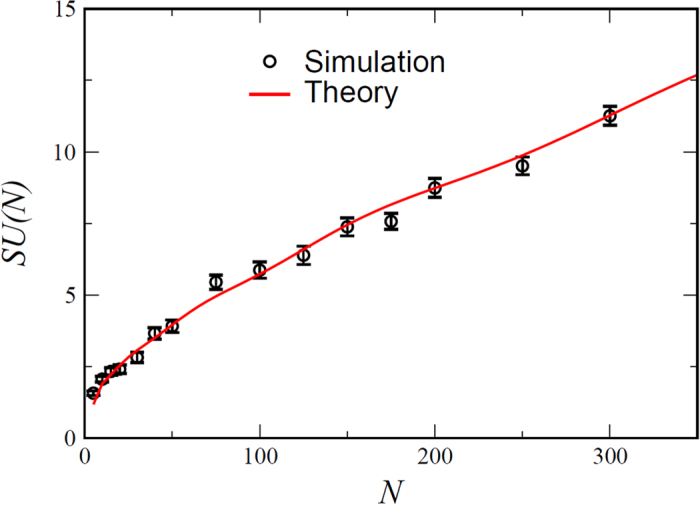
The number of SU states as a function of *N* for the asymmetric zero-infinity model (note the linear scale, in contrast to Fig. 2. Results were obtained from an average over simulations of random networks with 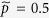, *N* running from 5 to 300. Points correspond to the number of SU states in a single realization, full line is the exact sum over *S* of (4). The asymptotic relationship (5) converges to this sum very slowly, see SI section II.

Here 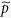 is the chance that a randomly chosen *c*_*i*,*j*_ = 0, which leads to a factor of 2 in the exponent as compared to the symmetric case where *p* is the chance that both *c*_*i*,*j*_ = 0 and *c*_*j*,*i* _= 0. The last factor, 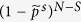, reflects the condition that none of the other *N* − *S* nodes has a directed dominance links to all the clique member, meaning that all other nodes are inferior with respect to at least one species in the clique. This seemingly minor modification changes the asymptotic growth mode from superpolynomial to sublinear:


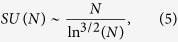


so the conclusion drawn in the symmetric case is relevant, a fortiori, for asymmetric communities: there is no exponential growth in the number of *SU*s with *N*, and consequently no niche-neutral transition at large *N*. The derivation of Eq. (5) from (4) is explained in section II of the SI and the agreement between the results and numerical simulations is demonstrated in Fig. 3.

Another important result derived in the SI is an expression for S^*^, the typical number of species in a single SU state, i.e., the maximum of *SU*(*N, S*). For both the symmetric model and the asymmetric model we obtain,


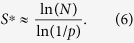


Accordingly, for *N* a few hundreds and *p* values that are not vanishingly small, the typical number of species in a single SU is 5–8. This estimate agrees with the results of our simulations of the network model and with simulations of the Lotka-Volterra dynamics, Eq. (1), to which we turn in the next paragraph. It implies that in the regime of strong enough competition, such that the system supports alternating steady states (see Ref. 26), it is hard to find such a state with more than, say, 8 species for reasonable values of *N*.

**Figure 4:**
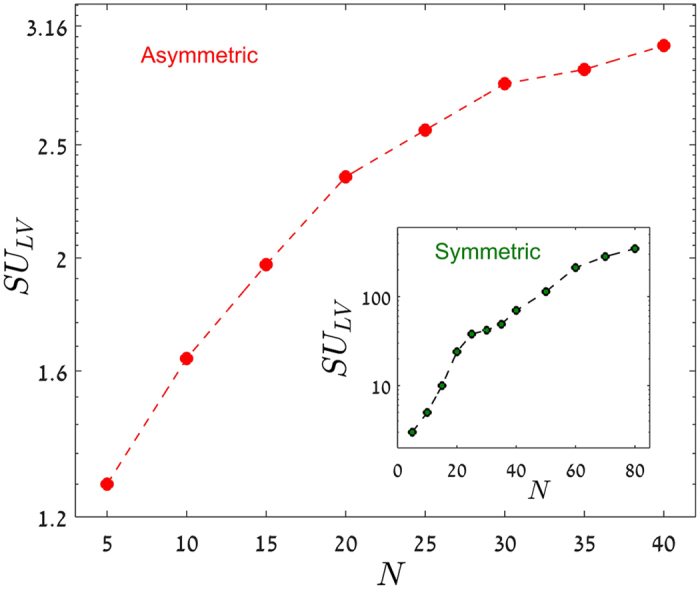
*SU*_*LV*_ is the average number of SU states for a Lotka-Volterra system (Eq. 1) with continuous *c*_*i*,*j*_ drawn from a Gamma distribution with 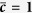 and *σ*^2^ = 1. For *N* ≤ 20 the number of states has been obtained from a comprehensive survey of all 2^*N*^ possible combinations, while for *N* > 20 SU’s were identified by integrating Eq. 1, from random initial conditions, until it reaches a SU state, and iterating this scheme 200000 times. In the main panel the results are presented for the asymmetric case, while the inset shows the results for the symmetric case. In both cases the subexponential growth of the number of *SU*_*LV*_ with *N* is manifested, and the theoretical predictions for the zero-infinity limit [Eqs (3) and (5)] are way above the numbers obtained here (see supplementary). While up to *N* = 20 the symmetric case appears to grow exponentially as seen in Ref. 21, above this value the graph turns over.

The model considered here is an extreme case of a generic competition system, where the interaction matrix may be replaced by a zero-one adjacency matrix. We see no reason to believe that the generic system with finite *c*_*i*,*j*_s falls into a different equivalence class. Actually our numerics for the case where the *c*_*i*,*j*_s are picked from a Gamma distribution indicates a sub-exponential growth in the number of SUs even for symmetric Lotka-Volterra matrices with continuously distributed *c*_*i*,*j*_s (see Fig. 4), and suggest that the number of SU’s obtained in Eqs (3) and (5) is an upper bound for the number of SU’s of generic interaction matrices (unless the *c*_*i*,*j*_s are vanishingly small, see discussion).

To underscore these statements and to bridge the gap between the zero/infinity limit and the standard generalized Lotka-Volterra system with continuous *c*_*i*,*j*_, an intermediate model is presented in the methods section and analyzed in section III of the SI. In this intermediate model *c*_*i*,*j*_s are either zero or take a finite value *A*, so the competition matrix elements have finite mean and variance, allowing for a fair comparison with the continuous case. This “binary” model is shown analytically to reproduce exactly the results of the zero/infinity model for sufficiently strong interactions for finite *N*. Numerical simulations show that indeed the number of SU’s is an upper bound to the continuous Lotka-Volterra system considered in Refs 21, 22 and 26.

## Community Dynamics

Until now we have focused on the identification, and counting, of the number of SU’s in different situations. This, however, is not enough to specify the behavior of a local community coupled to a regional pool. An SU is stable under the deterministic dynamics (1), but when noise and disturbances (which are almost always quite strong in ecosystems) are added to the model, local populations may go extinct and new species may invade^30^, leading to a modification of the overall strength of the local competition with respect to the regional interaction. Moreover, an asymmetric system may support, beyond SU states, other attractive manifolds like limit cycles and chaotic attractors. A simple example is that of a three species system with “rock-paper-scissors” circular dynamics, species 1 invades 2 but is dominated by 3 and so on^26^. To understand better the dynamical aspects of community assembly we have simulated the system deterministic dynamics starting from random initial conditions. This allows us to find out the duration of the transient until the system reaches an SU, and to see if the system enters a periodic/chaotic orbit.

**Figure 5:**
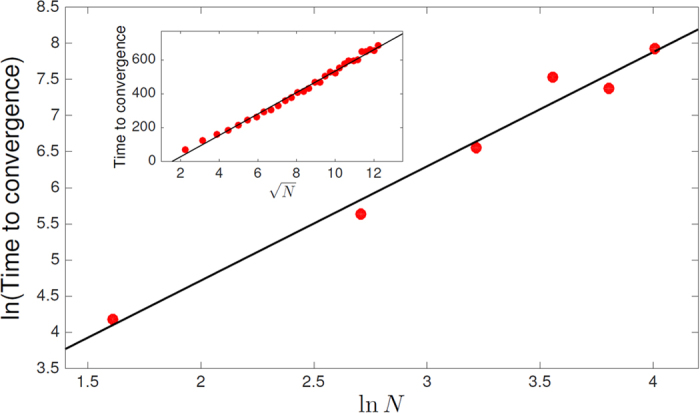
**ln (Convergence time) vs. In** ***N*****, the species richness of the regional community.** The dynamics of Eq. 1 was simulated, with *c*_*i*,*j*_s that were picked at random from a Gamma distribution with mean 0.9 and variance 5/*N* (variance should scale with 1/*N* to keep the overall competition stress independent of *N*). Points represent the time it take this system to converge to an SU from random initial conditions. For the asymmetric case (main panel, each point reflects an average over 65 runs each with different random competition matrix) the time to convergence grows like *N*^1.56^ (thick black line) while for the symmetric case (each point is the average over 2000 runs) the same numerical experiment yields a 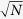 dependence.

Our simulations indicate that the periodic/chaotic orbits associated with the asymmetric dynamics are rare in the large *N* limit, and almost all initial conditions eventually arrive at an SU state. However, the symmetric and the asymmetric systems differ dramatically in the convergence time, as demonstrated in Fig. 5. In symmetric systems the convergence time grows like 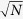, while it grows faster than *N*^3/2^ in the asymmetric case. This feature reflects the presence of many “almost attractive” orbits and long excursions in the asymmetric system. Accordingly, for a symmetric system one may expect that the dynamics is dominated by long periods in which the system is trapped in a single SU, with ecological regime shifts that drive it from one state to another as suggested in Ref. 19. In the asymmetric system, on the other hand, one expects that under the effect of disturbances (that kick the system out of an SU and send it on a long excursion) the relevance of the SUs becomes negligible and the system is in the intermittent phase (or, in terms of Ref. 31, alternative transient states) that was demonstrated in Ref. 26.

## Discussion

Community dynamics is the arena on which the evolutionary process unfolds. Darwin’s theory of natural selection and the survival of the fittest suggest a mechanism that governs the evolutionary dynamics and the origin of species, but at the same time it makes difficult the task of explaining the spectacular species richness observed in natural communities. Are there so many “fittest” species around?

Some researchers believe that the answer for this question is indeed positive: that each of the millions of species observed in nature is superior to its competitors with respect to a certain niche. Others consider this hypothesis as implausible, in particular when the number of different resources appear to be small. Moreover, May’s complexity-diversity puzzle implies that a community with substantial niche overlap will collapse, meaning that an almost complete niche-separation is needed to explain the coexistence of any species.

Given that, a lot of attention has been given in the last decades to the patterns observed in local communities, recognized as the elementary building blocks of the system. The dynamics in these communities is affected by local processes such as competition, predation and symbiosis, by migration of species from the regional pool and by stochastic and random effects.

In a recent work^26^ we have tried to classify the dynamics of a stochastic local community of competing species (in the asymmetric case) along two different axes: the overall strength of competition (average niche overlap, 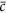) and the fitness differences *σ*^2^. When 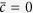 all species are non-interacting, as suggested in McArthur-Wilson model, while if 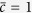 and *σ* = 0 all individuals are equal and the system is described by Hubbell’s neutral theory^32^. Between these two extremes we have found regimes of full and partial coexistence (when 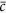 is relatively small), regimes of alternate stable states when 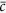 is large, and in between we observed a region of parameters where the system fails to relax to an SU. Instead, in this regime the dynamics is intermittent, where the community structure changes dramatically over time and the instantaneous assembly is usually invadable. As noted above, this intermittent phase appears to be related to the long convergence times demonstrated in Fig. 5, combined with the effect of constant perturbations, like the demographic noise in Ref. 26.

A priori, we cannot see a reason to prefer an explicit dynamical model, like the Lotka-Volterra equations (1), over the zero-infinity dynamics considered in this paper or its counterpart, the binomial model, presented in the SI. The actual dynamics of interspecific competition is very complex, affected by many factors, and there appears to be no way to map it into a realistic model with a reasonable number of parameters, not to mention the inference of the values of these parameters from empirical datasets. Both the LV system and the network model considered here aim at providing a qualitative picture, explaining the generic characteristics of systems in which species compete, proliferate, migrate and go extinct. We believe that the network analogy is appropriate as long as *σ* is large and 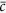 is not too small, which is the most interesting regime. As explained in the supplementary, the network analogy fails when 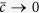 (where all the *N* species may occupy every local patch, the full/partial coexistence phase in the language of^26^) or when *σ* → 0 (the limit corresponding to the neutral or neutral-like behavior, where the system is governed by noise, see Ref. 33). Experimental studies (see, e.g., Refs 34 and 35) also suggest that the *c*_*i*,*j*_s are *O*(1) (i.e., interspecific competition does not differ substantially in magnitude from intraspecific competition) and that the variance is quite large. We believe that the glass transition results of Ref. 22 mentioned above, with the assumption that the number of SUs grow exponentially with *N*, are valid in the opposite limit where both the mean and the variance of the *c*_*i*,*j*_s scale like 1/*N* (see Ref. 36).

Finally, we believe that the geometric approach presented here may be extended to include more complicated networks. In particular, a foodweb system, like the one considered in Ref. 37 has a few levels (primary producers, predators, top predators etc.), within each of which different species compete, but the top levels depend on consuming individuals from lower levels. Similarly, in a plant-pollinator and other mutualistic network like those considered in Ref. 38, the two networks are interdependent. In both cases the theory of dependent networks^39^ may be relevant. The effect of different architectures, like the modular structure found in food webs^40^ and the nested structure associated with mutualistic networks^41^,^42^ is also an interesting factor, and one would like to study this is the context of network dynamics. We hope to address these topics in subsequent publications.

## Methods

Our aim is to find the number of SU states for a community of competing species described by the generalized Lotka-Volterra equation^1^. Commonly the *c*_*i*,*j*_s are drawn independently from a uniform, positive semi-definite, distribution with a given mean and variance^22^,^26^. A *c*_*i*,*j*_ matrix (for simplicity the examples are given for the symmetric case) may look like,


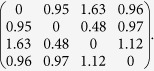


Since we used a Gamma distribution in our simulations, we denote this as the Gamma model.

The mapping of this model to the maximum clique problem (which is clearly exact in the limit where all matrix elements are either zero or infinite) involves two steps of simplification. First we assume that all the elements of the *c*_*i*,*j*_ matrix either are strictly zero or are equal to a finite constant *C *·* A*, so the interaction matrix takes a form, say,


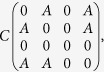


where we introduced two constants, *C* and *A*, to distinguish between the overall strength of the competition and the matrix structure. In the SI (section III) we show how these constants should scale such that this “binary” model and the continuum Gamma model matrix elements will have the same mean and variance.

In the binary model every maximal clique is (trivially) stable, since the strength of competition between all species in such a clique is zero. However, if *A* is too small such a maximal clique may be invadable by one of the species outside the clique. In section III of the SI we show that this cannot happen if *C* is large enough (the condition is *C* > 1 − *p*, where *p* is the fraction of zeros in the competition matrix). Above this critical *C* our maximum cliques are both stable and uninvadable, and one may replace the zero-*A* interaction matrix by the corresponding zero/infinity matrix,


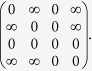


Moreover, we provide in SI III numerical evidence to show that the number of cliques of this binomial model is an upper bound for the continuum competition (Gamma) model with the same mean and variance.

## Additional Information

**How to cite this article**: Fried, Y. *et al*. Communities as cliques. *Sci. Rep.* **6**, 35648; doi: 10.1038/srep35648 (2016).

## Supplementary Material

Supplementary Information
