# Supplementary material for: Communities as cliques
Source: Sci Rep. 2016 Oct 19;6:35648. doi: 10.1038/srep35648 (PMC5069479; doi:10.1038/srep35648)
Supplement: Supplementary Information [file srep35648-s1.pdf]

Supplementary information to: Communities as cliques

Yael Fried, David A. Kessler and Nadav M. Shnerb
Department of Physics, Bar-Ilan University, Ramat-Gan IL52900, Israel.

This supplementary material consists of three sections. In the first two sections we provide the mathematical details of the derivation of Eq. (3) of the main text from Eq. (2), and the derivation of Eq. (5) from Eq. (4), respectively. The aim of the third section is to analyze an intermediate model presented in the method section of the main text. This model bridges between our infinite σ model and the standard generalized Lotka-Volterra system (Eq. (1) of the main text with $c_{i,j}$ that are drawn from a continuous distribution with finite moments).

I. ASYMPTOTICS OF $SU(N)$, SYMMETRIC NETWORK

Let p be the probability of a symmetric coexistence link ($c_{i,j} = c_{j,i} = 0$). Then the number of maximal cliques of size S in a random graph of N nodes is given in Eq. (2) of the main text, following [1]:

$$SU(N, S) = \binom{N}{S} p^{S(S-1)/2}(1 - p^S)^{N-S} \tag{1}$$

To get the large N asymptotic of this sum we define

$$\alpha \equiv \ln(1/p). \tag{2}$$

Using Stirling's formula we can approximate (1) for large N, $S \ll N$, as

$$\ln SU(N, S) \approx S \ln N - (S + 1/2)\ln(S) + S - \frac{1}{2}\ln(2\pi) - \alpha S(S-1)/2 - (N - S)e^{-\alpha S}. \tag{3}$$

Taking a derivative with respect to S, we get an equation for S^*, the value of S that gives the maximal contribution to the sum in Eq. (1):

$$\begin{aligned} 0 &= \ln N - \ln S^* - 1/(2S^*) - \alpha(S^* - 1/2) + \tilde{p}^{-S^*} + \alpha e^{-\alpha S^*}(N - S^*) \\ &= \alpha N e^{-\alpha S^*} + \ln(N e^{-\alpha S^*}) - \ln S^* - 1/(2S^*) + \frac{1}{2}\alpha + e^{-\alpha S^*} - \alpha S^* e^{-\alpha S^*} \end{aligned} \tag{4}$$

The dominant balance is between the terms $\alpha N e^{-\alpha S^*}$ and $-\ln S^*$, giving

$$\alpha N e^{-\alpha S^*} = \ln S^* \tag{5}$$

so that

$$S^* \approx \frac{\ln N}{\alpha} + \mathcal{O}(\ln(\ln(\ln(N)))). \tag{6}$$

As a first approximation one may assume that the logarithm of the sum (1) is equal to the contribution from S^* alone. Plugging this into Eq. (3) one finds,

$$\ln SU(N) \approx \ln SU(N, S^*) \approx \frac{\ln^2 N}{2\alpha} - \frac{\ln N}{\alpha}\ln\left(\frac{\ln N}{e\alpha}\right). \tag{7}$$

In principle, we have to include also the contribution from a Gaussian integral in $S - S^*$ around S^*. However, the coefficient of $(S - S^*)^2$ in this integral is given by,

$$\frac{d^2}{dS^2}\ln SU(N, r)\Big|_{r=S^*} \approx -N\alpha^2 e^{-\alpha S^*} \approx \ln S^* \alpha \approx \alpha \ln\left(\frac{\ln N}{\alpha}\right) \tag{8}$$

so in fact the correction decays so quickly around S^* that only the leading term (7) contributes (note that the sum is discrete). This leads to Eq. (3) of the main text.

II. ASYMPTOTICS OF $SU(N)$, ASYMMETRIC NETWORK

For asymmetric links, we found that (Eq. (4) of the main text)

$$SU(N,S) = \binom{N}{S} \tilde{p}^{S(S-1)} (1-\tilde{p}^S)^{N-S} \tag{9}$$

Again, writing things in terms of $\alpha \equiv \ln(1/\tilde{p})$, we have

$$\ln SU(N,S) \approx S \ln N - (S+1/2)\ln(S) + S - \frac{1}{2}\ln(2\pi) - \alpha S(S-1) - (N-S)e^{-\alpha S} \tag{10}$$

The dominant S, S^*, obeys

$$\begin{aligned} 0 &= \ln N - \ln S^* - 1/(2S^*) - \alpha(2S^*-1) + \tilde{p}^{-S^*} + \alpha e^{-\alpha S^*}(N - S^*) \\ &= \alpha N e^{-\alpha S^*} + \ln(Ne^{-\alpha S^*}) - \alpha S^* - \ln S^* - 1/(2S^*) + \alpha + e^{-\alpha S^*} - \alpha S^* e^{-\alpha S^*} \end{aligned} \tag{11}$$

The dominant balance is now between the terms $\alpha N e^{-\alpha S^*}$ and $-\alpha S^*$, giving

$$S^* \approx \frac{\ln N}{\alpha} - \frac{\ln\left(\frac{\ln N}{\alpha}\right)}{\alpha} \tag{12}$$

Using this, the $\mathcal{O}(\ln^2 N)$ terms cancel, leaving us with

$$\ln SU(N) \approx SU(N,S^*) \approx \ln N - \frac{3}{2}\ln\left(\frac{\ln N}{\alpha}\right) - \frac{1}{2}\ln 2\pi, \tag{13}$$

and this yields the result that appears in Eq. (5) of the main text. Note that the convergence of this expression to the exact sum over S in Eq. (9) is quite slow and nonmonotonic, see Figure 1.

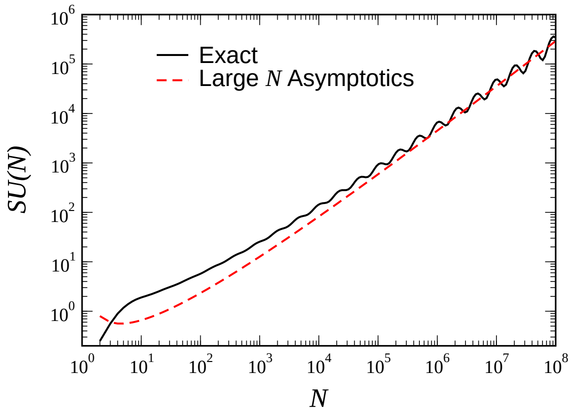

FIG. 1: The number of SUs in the asymmetric case: a comparison between the exact sum over S in Eq. (9) (black solid line) and the asymptotic formula (13) (dashed red line).

Again, the decay of $SU(N,S)$ is so rapid,

$$\left.\frac{d^2}{dS^2} \ln SU(N,r)\right|_{r=S^*} \approx -N\alpha^2 e^{-\alpha S^*} \approx -\alpha S^* \approx -\frac{\ln N}{\alpha} \tag{14}$$

that only the S^* term contributes to the sum asymptotically.

III. THE BINARY MODEL

In the main text we discussed the connection between SUs and cliques in the limit where the competition matrix elements are either zero or infinite, so two species i and j may be noninteracting ($c_{ij} = c_{ji} = 0$), mutually exclusive ($c_{ij} = \infty,\ c_{ji} = \infty$) or in dominance relationships ($c_{ij} = \infty,\ c_{ji} = 0$ or $c_{ij} = 0,\ c_{ji} = \infty$). This presentation leads us immediately to the notion of cliques and to the conclusions we drow from the equivalence between cliques and SUs. Here we would like to discuss the relationships between this extreme limit and the "standard" GLV description of the system, in which the c_{ij}s are picked at random from a continuous distribution with finite moments. To be specific we will use, as in [2], Gamma distributed $c_{i,j}$s and denote this version of the generalized Lotka-Volterra as the Gamma model.

To bridge the gap between the zero-infinite and the Gamma model, we present here an intermediate scenario, the binary model, which allows us to obtain a few analytic results that establish the relevance of the clique-based analysis. At the same time, this model, which has finite σ and C, facilitates a numerical comparison with the standard GLV model. These three types of interaction matrices - Gamma, binary and zero-infinite - are illustrated in Figure 2. For simplicity our discussion is presented for the symmetric case, but the results are general.

Gamma Binary *zero/infinity*

$$\begin{pmatrix} 0 & 0.95 & 1.63 & 0.96 \\ 0.95 & 0 & 0.48 & 0.97 \\ 1.63 & 0.48 & 0 & 1.12 \\ 0.96 & 0.97 & 1.12 & 0 \end{pmatrix} \qquad C\begin{pmatrix} 0 & A & 0 & A \\ A & 0 & 0 & A \\ 0 & 0 & 0 & 0 \\ A & A & 0 & 0 \end{pmatrix} \qquad \begin{pmatrix} 0 & \infty & 0 & \infty \\ \infty & 0 & 0 & \infty \\ 0 & 0 & 0 & 0 \\ \infty & \infty & 0 & 0 \end{pmatrix}$$

FIG. 2: Three types of interaction matrices $c_{i,j}$. The left matrix corresponds to a four species community in which the niche overlap between species is assumed to be a random number taken from a Gamma distribution. To the right one sees an interaction matrix that corresponds to the zero/infinity limit considered in the main text, where every two species are either non-interacting or are mutually exclusive. The interaction matrix in the middle (C and A are constants, see below) exemplifies our binary model: like the infinite σ case is has only two types of interactions, but unlike it, it admits a finite value for σ, allowing for a comparison with GLV systems with continuous $c_{i,j}$s that have the same parameters. For simplicity we present examples for the symmetric case, where the level of competition between every two species is characterized by a single number that corresponds to the niche overlap between these two species. In the non-symmetric case $c_{ij} \neq c_{ji}$, but all other features of the three models are the same.

To begin, let us rewrite the GLV equation used in the main text,

$$\frac{dx_i}{dt} = x_i - x_i \left(x_i + C \sum_{j \neq i}^{N} c_{i,j} x_j \right). \tag{15}$$

where now (following [2]) we assume that the $c_{i,j}$s are normalized such that their average is unity and C sets the overall scale of the interaction. In the binary model, the $c_{i,j}$s are either A or zero, so to fix their average to unity we must choose $A = 1/(1-p)$, where p is the probability of $c_{i,j} = c_{j,i} = 0$. It follows that the variance of the $c_{i,j}$s is

$$\mathrm{Var}[c_{i,j}] = \sigma^2 = A^2 p(1-p) = \frac{p}{1-p}. \tag{16}$$

Thus, our binary model allows us to control, as in the standard Gamma distribution model, both the overall competition strength C and the variance of the competition, σ^2, and to compare Gamma and binary competition matrices with the same mean and variance.

The binary model is similar in many respects to the infinite σ model discussed in the main text. Indeed, its maximal cliques are *precisely* the SUs for C greater than some threshold value $C_t < 1$. It is trivial to see that a maximal clique is a solution of the GLV equation (15), since for that subset of species the model is noninteracting, and the state with $x_i = 1$ for all species i represented in the maximal clique is feasible and stable. The nontrivial task is to find under what conditions the solution is also uninvadable, rendering it an SU. The equation of invadability for an absent species α is

$$\dot{x}_\alpha = x_\alpha \left[1 - C \sum_{i \in \boldsymbol{\mathcal{S}}} c_{\alpha,i} \right], \tag{17}$$

where the sum is over all present species $i \in$ clique. In the binary model $c_{i,j}$ is either A or zero, so $\sum_{i \in \boldsymbol{\mathcal{S}}} c_{\alpha,i}$ is simply A times the integer $m_\alpha^{\boldsymbol{\mathcal{S}}}$, the number of species (in the clique $\boldsymbol{\mathcal{S}}$) with nonzero $c_{\alpha,i}$ element with the spececies α. Thus, the solution is uninvadable (and so is an SU) as long as,

$$C < \max_\alpha \frac{1}{\sum_{i \in \boldsymbol{\mathcal{S}}} c_{\alpha,i}} \qquad \text{or} \qquad C < \max_\alpha \frac{1}{A m_\alpha^{\boldsymbol{\mathcal{S}}}} \tag{18}$$

the maximum is taken from the values for all the species α which are *not* in the clique. Since any species out of the clique has at least one enemy in the clique (otherwise, the clique is not maximal) for any $C > 1 - p$ no clique is invadable.

For example, let us consider the following competition matrix:

$$C \begin{pmatrix} 0 & 0 & 0 & 6 \\ 0 & 0 & 0 & 0 \\ 0 & 0 & 0 & 0 \\ 6 & 0 & 0 & 0 \end{pmatrix}.$$

This is a 4-species realization of the binary model, and since there are 6 possible competition terms from which only one is active, it follows that $p = 5/6$ hence $A = 6$.

Clearly, species $\{1, 2, 3\}$ constitute a maximum clique: they all do not compete with each other, while species $\{4\}$ suffers from competition with $\{1\}$. Eq. 15 for x_4 (when the island is occupied by species $\{1, 2, 3\}$, each with abundance one) now reads,

$$\frac{dx_4}{dt} = x_4 - x_4^2 - 6Cx_1x_4 = (1 - 6C)x_4 - x_4^2, \tag{19}$$

so x_4 may invade (its linear growth term is positive) only if $C < 1/6$, but above this value the maximal clique is indeed an SU. This agrees with Eq. (18) as $A = 6$ and $m_4^{\boldsymbol{\mathcal{S}}} = 1$.

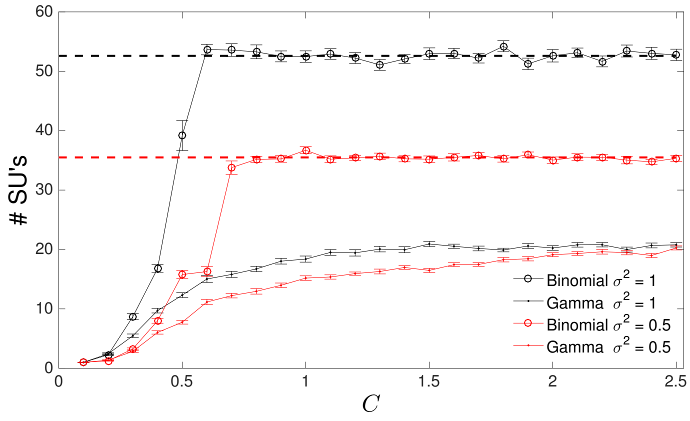

FIG. 3: The number of SU states as a function of C for the binary symmetric model and for the Gamma model, both with $N = 20$. Graphs show the average and the standard deviation (error bars) of the number of SUs for random matrices with $\sigma^2 = 0.5$ and $\sigma^2 = 1$. For the binary model one can clearly see the saturation above some C_t, where all the maximal cliques are SUs and further increase in the value of C has no effect. The value of C_t decreases as σ^2 (and p) increases, in agreement with our predictions. The dashed lines are the expected number of maximal cliques, computed from Eq. (2) of the main text with the relevant values of p (1/2 and 1/3), and thus are the number of SUs in the infinite-σ model with the same value of p. Clearly, the number of SU saturates to these values, which are way above N. For the Gamma model, on the other hand, the maximum number of SUs is about N, and one can see that the number of SUs in the binary model is always an upper bound to the number of SUs in the Gamma model. Results were obtained by averaging over 65 different matrices in each case.

However, the condition $C > 1 - p$ turns out to be too restrictive for large systems. On average, not only one but a fraction $1 - p$ of the $c_{\alpha,i}$ are nonzero, so a naive estimate of the threshold C, C_t, is

$$C_t = 1/(S(1 - p)A) = 1/S,$$

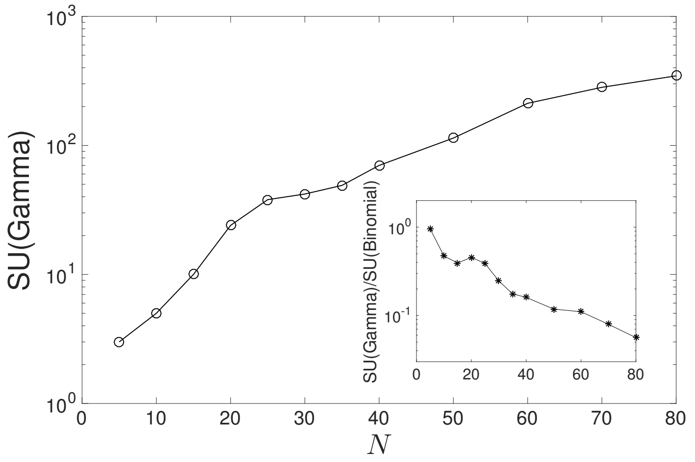

FIG. 4: The number of SUs for the Gamma distributed, symmetric Lotka-Volterra model, as shown in Fig. 4 of the main text, is reproduced in the main panel. The inset shows these numbers divided by the number of cliques in the binary model with the same values of $C = 1$ and σ. One realizes that the number of SUs obtained for the binary model provides an upper bound for the Gamma distributed $c_{i,j}$ case. Since for this value of C the number of SUs in the binary model is precisely the number of maximal cliques of the infinite-σ model, which we have shown to be subexponential, this is a clear indication that the Gamma distributed Lotka-Volterra model behaves likewise.

where S is the size of the maximal clique. Since the typical size of a clique grows (slowly) with N, we expect that $C_t \to 0$ as $N \to \infty$, and so our clique picture should become exact in that limit - *any maximal clique of the infinite-σ model will be an SU of the corresponding binary system.* We have verified these calculations by comparing the set of maximal cliques and SUs for the binary model and seeing that indeed the two sets are identical down to some threshold C satisfying our bound.

Within the binary model, the general features of the Gamma distribution model are preserved. For example, if C is smaller than some threshold value $C < C_1$ (using the notations defined in fig 1 of [2]) there is exactly one SU, namely the state with all species present. This is clearly not a maximal clique for general p. Then, up to a second critical point C_2, there is still only a single SU, with however, some species are missing. The general trend is that below C_t the number of SUs falls below the number of maximal cliques, and decreases monotonically as C decreases. This is demonstrated in Fig. 3, where we have plotted the number of SU's in the binary model (averaged over a number of realizations) as a function of C for $N = 20$ and two different values of σ.

Together with the results of the binary model we have plotted the average number of SUs in the Gamma distribution model for the same values of N, C and σ. One sees that the number of SUs in the Gamma distribution model is significantly smaller. In the limit $C \to \infty$ every pair of species in the Gamma model is mutually exclusive so every species is an SU, meaning that the number of SUs in the Gamma model approaches N for large C. This numerical evidence, together with those presented in Fig. 4 of the main text and in Fig. 4 here, reinforce our claim that the number of SUs in the Gamma distribution case also grows slower than exponentially with N.

[1] Bollobás B, Erdös P (1976) Cliques in random graphs. *Mathematical Proceedings of the Cambridge Philosophical Society* 80(3):419–427.
[2] Kessler DA, Shnerb NM (2015) Generalized model of island biodiversity. *Physical Review E* 91(4):042705.
